# Supplementary material for: Examination of hyper-palatable foods and their nutrient characteristics using globally crowdsourced data
Source: PLoS One. 2025 Jun 6;20(6):e0325479. doi: 10.1371/journal.pone.0325479 (PMC12143524; doi:10.1371/journal.pone.0325479)
Supplement: S1 File — S1 Table. Prevalence of HPF within Food Main Categories across Countries. S2 Table. Summary of the Prevalence of HPF across Countries.S3 Table. Logistic regression results for the HPF prevalence compared to United States. S4 Table. Logistic regression results for the FSOD prevalence compared to United States. S5 Table. Logistic regression results for the FS prevalence compared to United States. S6 Table. Logistic regression results for the CSOD prevalence compared to United States. S7 Table. Descriptive Statistics of Nutritional Compositions of FSOD across Countries.S8 Table. Descriptive Statistics of Nutritional Compositions of FS across Countries. S9 Table. Descriptive Statistics of Nutritional Compositions of CSOD across Countries. S10 Table. Ordered Beta Regression Results for nutritional compositions of FSOD across countries compared to United States.S11 Table. Ordered Beta Regression Results for nutritional compositions of FS across countries compared to United States. S12 Table. Ordered Beta Regression Results for nutritional compositions of CSOD across countries compared to United States. S13 Table. Distinct and Overlapping percentage of HPF and UPF across countries. (ZIP) [file pone.0325479.s001.zip › S1 Table.docx]

| **S1 Table. Prevalence of HPF within Food Main Categories across Countries** | | | | |
| --- | --- | --- | --- | --- |
| **Country** | **Food Main Category** | **N of Food Items** | **N of HPF** | **Proportion of HPF (%)** |
| United States | Salty snacks | 4443 | 3630 | 81.70 |
|  | Composite foods | 14689 | 11300 | 76.93 |
|  | Sugary snacks | 38706 | 28377 | 73.31 |
|  | Milk and dairy products | 18699 | 13110 | 70.11 |
|  | Fats and sauces | 17800 | 11346 | 63.74 |
|  | Cereals and potatoes | 25669 | 15508 | 60.42 |
|  | Fish‚ Meat‚ Eggs | 15556 | 8836 | 56.80 |
|  | Fruits and vegetables | 13279 | 1708 | 12.86 |
| France | Salty snacks | 5764 | 4949 | 85.86 |
|  | Milk and dairy products | 8516 | 6282 | 73.77 |
|  | Composite foods | 10826 | 7572 | 69.94 |
|  | Sugary snacks | 19207 | 13063 | 68.01 |
|  | Fish‚ Meat‚ Eggs | 8064 | 4925 | 61.07 |
|  | Fats and sauces | 5247 | 2655 | 50.60 |
|  | Cereals and potatoes | 18628 | 7963 | 42.75 |
|  | Fruits and vegetables | 5218 | 1071 | 20.53 |
| Italy | Composite foods | 1095 | 947 | 86.48 |
|  | Salty snacks | 440 | 374 | 85.00 |
|  | Milk and dairy products | 1107 | 734 | 66.31 |
|  | Sugary snacks | 4336 | 2822 | 65.08 |
|  | Fish‚ Meat‚ Eggs | 892 | 546 | 61.21 |
|  | Fats and sauces | 568 | 299 | 52.64 |
|  | Cereals and potatoes | 6213 | 2508 | 40.37 |
|  | Fruits and vegetables | 475 | 96 | 20.21 |
| Germany | Composite foods | 1711 | 1383 | 80.83 |
|  | Salty snacks | 860 | 692 | 80.47 |
|  | Milk and dairy products | 1155 | 860 | 74.46 |
|  | Fish‚ Meat‚ Eggs | 739 | 489 | 66.17 |
|  | Sugary snacks | 2496 | 1607 | 64.38 |
|  | Fats and sauces | 1223 | 606 | 49.55 |
|  | Cereals and potatoes | 5260 | 1978 | 37.60 |
|  | Fruits and vegetables | 854 | 207 | 24.24 |
| Spain | Salty snacks | 968 | 784 | 80.99 |
|  | Composite foods | 924 | 704 | 76.19 |
|  | Milk and dairy products | 1197 | 826 | 69.01 |
|  | Fish‚ Meat‚ Eggs | 1003 | 656 | 65.40 |
|  | Sugary snacks | 3545 | 2308 | 65.11 |
|  | Cereals and potatoes | 3678 | 1863 | 50.65 |
|  | Fats and sauces | 824 | 395 | 47.94 |
|  | Fruits and vegetables | 843 | 198 | 23.49 |
| Switzerland | Composite foods | 1163 | 985 | 84.69 |
|  | Salty snacks | 575 | 479 | 83.30 |
|  | Milk and dairy products | 1422 | 1089 | 76.58 |
|  | Sugary snacks | 1960 | 1346 | 68.67 |
|  | Fish‚ Meat‚ Eggs | 781 | 519 | 66.45 |
|  | Fats and sauces | 762 | 434 | 56.96 |
|  | Cereals and potatoes | 2617 | 1268 | 48.45 |
|  | Fruits and vegetables | 448 | 105 | 23.44 |
| Belgium | Salty snacks | 553 | 476 | 86.08 |
|  | Milk and dairy products | 824 | 617 | 74.88 |
|  | Fish‚ Meat‚ Eggs | 607 | 420 | 69.19 |
|  | Sugary snacks | 1638 | 1098 | 67.03 |
|  | Composite foods | 808 | 538 | 66.58 |
|  | Fats and sauces | 593 | 357 | 60.20 |
|  | Cereals and potatoes | 1851 | 788 | 42.57 |
|  | Fruits and vegetables | 433 | 112 | 25.87 |
| United Kingdom | Salty snacks | 583 | 496 | 85.08 |
|  | Milk and dairy products | 646 | 479 | 74.15 |
|  | Sugary snacks | 1520 | 1069 | 70.33 |
|  | Fish‚ Meat‚ Eggs | 694 | 370 | 53.31 |
|  | Composite foods | 1260 | 633 | 50.24 |
|  | Cereals and potatoes | 1692 | 812 | 47.99 |
|  | Fats and sauces | 615 | 292 | 47.48 |
|  | Fruits and vegetables | 286 | 73 | 25.52 |
| Canada | Composite foods | 394 | 298 | 75.63 |
|  | Salty snacks | 369 | 272 | 73.71 |
|  | Milk and dairy products | 657 | 475 | 72.30 |
|  | Fish‚ Meat‚ Eggs | 465 | 317 | 68.17 |
|  | Sugary snacks | 1406 | 954 | 67.85 |
|  | Fats and sauces | 601 | 340 | 56.57 |
|  | Cereals and potatoes | 1127 | 564 | 50.04 |
|  | Fruits and vegetables | 321 | 57 | 17.76 |
| Netherlands | Salty snacks | 198 | 148 | 74.75 |
|  | Fish‚ Meat‚ Eggs | 158 | 114 | 72.15 |
|  | Composite foods | 189 | 134 | 70.90 |
|  | Milk and dairy products | 264 | 185 | 70.08 |
|  | Fats and sauces | 223 | 122 | 54.71 |
|  | Sugary snacks | 510 | 279 | 54.71 |
|  | Cereals and potatoes | 727 | 330 | 45.39 |
|  | Fruits and vegetables | 122 | 32 | 26.23 |
| Poland | Salty snacks | 169 | 147 | 86.98 |
|  | Composite foods | 104 | 84 | 80.77 |
|  | Milk and dairy products | 149 | 107 | 71.81 |
|  | Fish‚ Meat‚ Eggs | 66 | 47 | 71.21 |
|  | Sugary snacks | 341 | 220 | 64.52 |
|  | Fats and sauces | 135 | 52 | 38.52 |
|  | Fruits and vegetables | 95 | 36 | 37.89 |
|  | Cereals and potatoes | 547 | 201 | 36.75 |
| Ireland | Salty snacks | 101 | 85 | 84.16 |
|  | Milk and dairy products | 197 | 154 | 78.17 |
|  | Sugary snacks | 239 | 157 | 65.69 |
|  | Composite foods | 251 | 162 | 64.54 |
|  | Fish‚ Meat‚ Eggs | 145 | 76 | 52.41 |
|  | Fats and sauces | 158 | 79 | 50.00 |
|  | Cereals and potatoes | 445 | 199 | 44.72 |
|  | Fruits and vegetables | 62 | 15 | 24.19 |
| Portugal | Salty snacks | 102 | 84 | 82.35 |
|  | Sugary snacks | 402 | 300 | 74.63 |
|  | Fish‚ Meat‚ Eggs | 97 | 65 | 67.01 |
|  | Composite foods | 77 | 51 | 66.23 |
|  | Milk and dairy products | 164 | 81 | 49.39 |
|  | Fats and sauces | 39 | 18 | 46.15 |
|  | Cereals and potatoes | 495 | 216 | 43.64 |
|  | Fruits and vegetables | 55 | 17 | 30.91 |
| Bulgaria | Salty snacks | 84 | 77 | 91.67 |
|  | Composite foods | 43 | 39 | 90.70 |
|  | Fish‚ Meat‚ Eggs | 154 | 124 | 80.52 |
|  | Milk and dairy products | 321 | 252 | 78.50 |
|  | Sugary snacks | 247 | 166 | 67.21 |
|  | Fruits and vegetables | 76 | 38 | 50.00 |
|  | Cereals and potatoes | 218 | 101 | 46.33 |
|  | Fats and sauces | 91 | 42 | 46.15 |
| Australia | Salty snacks | 115 | 98 | 85.22 |
|  | Milk and dairy products | 105 | 67 | 63.81 |
|  | Sugary snacks | 267 | 164 | 61.42 |
|  | Cereals and potatoes | 344 | 170 | 49.42 |
|  | Fish‚ Meat‚ Eggs | 77 | 38 | 49.35 |
|  | Fats and sauces | 111 | 54 | 48.65 |
|  | Composite foods | 146 | 62 | 42.47 |
|  | Fruits and vegetables | 65 | 17 | 26.15 |
| Austria | Composite foods | 149 | 135 | 90.60 |
|  | Salty snacks | 108 | 97 | 89.81 |
|  | Milk and dairy products | 136 | 101 | 74.26 |
|  | Fish‚ Meat‚ Eggs | 58 | 40 | 68.97 |
|  | Sugary snacks | 215 | 146 | 67.91 |
|  | Fats and sauces | 76 | 36 | 47.37 |
|  | Fruits and vegetables | 45 | 15 | 33.33 |
|  | Cereals and potatoes | 390 | 129 | 33.08 |
| Mexico | Composite foods | 50 | 37 | 74.00 |
|  | Salty snacks | 92 | 65 | 70.65 |
|  | Milk and dairy products | 189 | 129 | 68.25 |
|  | Sugary snacks | 238 | 145 | 60.92 |
|  | Cereals and potatoes | 360 | 197 | 54.72 |
|  | Fats and sauces | 145 | 72 | 49.66 |
|  | Fish‚ Meat‚ Eggs | 71 | 27 | 38.03 |
|  | Fruits and vegetables | 29 | 5 | 17.24 |
