# Supplementary material for: Examination of hyper-palatable foods and their nutrient characteristics using globally crowdsourced data
Source: PLoS One. 2025 Jun 6;20(6):e0325479. doi: 10.1371/journal.pone.0325479 (PMC12143524; doi:10.1371/journal.pone.0325479)
Supplement: S1 File — S1 Table. Prevalence of HPF within Food Main Categories across Countries. S2 Table. Summary of the Prevalence of HPF across Countries.S3 Table. Logistic regression results for the HPF prevalence compared to United States. S4 Table. Logistic regression results for the FSOD prevalence compared to United States. S5 Table. Logistic regression results for the FS prevalence compared to United States. S6 Table. Logistic regression results for the CSOD prevalence compared to United States. S7 Table. Descriptive Statistics of Nutritional Compositions of FSOD across Countries.S8 Table. Descriptive Statistics of Nutritional Compositions of FS across Countries. S9 Table. Descriptive Statistics of Nutritional Compositions of CSOD across Countries. S10 Table. Ordered Beta Regression Results for nutritional compositions of FSOD across countries compared to United States.S11 Table. Ordered Beta Regression Results for nutritional compositions of FS across countries compared to United States. S12 Table. Ordered Beta Regression Results for nutritional compositions of CSOD across countries compared to United States. S13 Table. Distinct and Overlapping percentage of HPF and UPF across countries. (ZIP) [file pone.0325479.s001.zip › S12 Table.docx]

| **S12 Table. Ordered Beta Regression Results for nutritional compositions of CSOD across countries compared to United States** | | | | | |
| --- | --- | --- | --- | --- | --- |
| **Effects of countries on percentage of kcal from carbohydrate (PCARBK)** | | | | | |
| **Parameter** | **Odds-Ratio** | **SE** | **95% CI** | **z** | **p** |
| United States (reference) | 1.34 | 0.00 | (1.33, 1.35) | 98.45 | < .001 |
| France | 0.89 | 0.00 | (0.88, 0.90) | -21.19 | < .001 |
| Italy | 0.98 | 0.01 | (0.96, 1.00) | -1.93 | 0.053 |
| Germany | 0.91 | 0.01 | (0.89, 0.93) | -9.11 | < .001 |
| Spain | 0.94 | 0.01 | (0.92, 0.96) | -5.92 | < .001 |
| Switzerland | 0.91 | 0.01 | (0.89, 0.93) | -7.77 | < .001 |
| Belgium | 0.92 | 0.01 | (0.89, 0.94) | -5.78 | < .001 |
| United Kingdom | 0.89 | 0.01 | (0.87, 0.91) | -8.40 | < .001 |
| Canada | 1.05 | 0.02 | (1.01, 1.08) | 2.68 | 0.007 |
| Netherlands | 0.97 | 0.02 | (0.93, 1.02) | -1.12 | 0.264 |
| Poland | 0.96 | 0.03 | (0.91, 1.02) | -1.36 | 0.175 |
| Ireland | 0.92 | 0.03 | (0.87, 0.97) | -2.88 | 0.004 |
| Portugal | 0.93 | 0.03 | (0.88, 0.98) | -2.57 | 0.010 |
| Bulgaria | 1.11 | 0.05 | (1.02, 1.20) | 2.45 | 0.014 |
| Australia | 0.98 | 0.03 | (0.92, 1.04) | -0.60 | 0.545 |
| Austria | 0.85 | 0.03 | (0.79, 0.91) | -4.60 | < .001 |
| Mexico | 1.03 | 0.03 | (0.96, 1.09) | 0.82 | 0.411 |
|  | | | | | |

| **Supplemental Table S12. Ordered Beta Regression Results for nutritional compositions of CSOD across countries compared to United States (continued)** | | | | | |
| --- | --- | --- | --- | --- | --- |
| **Effects of countries on sodium per food weight (SODIpw)** | | | | | |
| **Parameter** | **Coefficient** | **SE** | **95% CI** | **z** | **p** |
| United States (reference) | 0.01 | 0.00 | (0.00, 0.00) | -1310.93 | < .001 |
| France | 0.92 | 0.01 | (0.91, 0.93) | -12.94 | < .001 |
| Italy | 0.99 | 0.01 | (0.97, 1.01) | -0.99 | 0.322 |
| Germany | 0.91 | 0.01 | (0.89, 0.94) | -7.19 | < .001 |
| Spain | 0.92 | 0.01 | (0.90, 0.95) | -6.36 | < .001 |
| Switzerland | 0.96 | 0.01 | (0.93, 0.99) | -2.77 | 0.006 |
| Belgium | 0.86 | 0.02 | (0.83, 0.90) | -7.61 | < .001 |
| United Kingdom | 0.80 | 0.01 | (0.77, 0.83) | -12.38 | < .001 |
| Canada | 0.98 | 0.02 | (0.95, 1.02) | -0.80 | 0.422 |
| Netherlands | 0.88 | 0.03 | (0.83, 0.93) | -4.21 | < .001 |
| Poland | 0.96 | 0.04 | (0.89, 1.03) | -1.13 | 0.260 |
| Ireland | 0.83 | 0.03 | (0.77, 0.90) | -4.73 | < .001 |
| Portugal | 0.81 | 0.03 | (0.75, 0.87) | -5.80 | < .001 |
| Bulgaria | 1.01 | 0.05 | (0.92, 1.11) | 0.18 | 0.859 |
| Australia | 0.89 | 0.04 | (0.83, 0.97) | -2.86 | 0.004 |
| Austria | 0.98 | 0.04 | (0.90, 1.07) | -0.38 | 0.704 |
| Mexico | 0.98 | 0.04 | (0.91, 1.06) | -0.49 | 0.627 |
