# Supplementary figures and images for: Examination of hyper-palatable foods and their nutrient characteristics using globally crowdsourced data
Source: PLoS One. 2025 Jun 6;20(6):e0325479. doi: 10.1371/journal.pone.0325479 (PMC12143524; doi:10.1371/journal.pone.0325479)

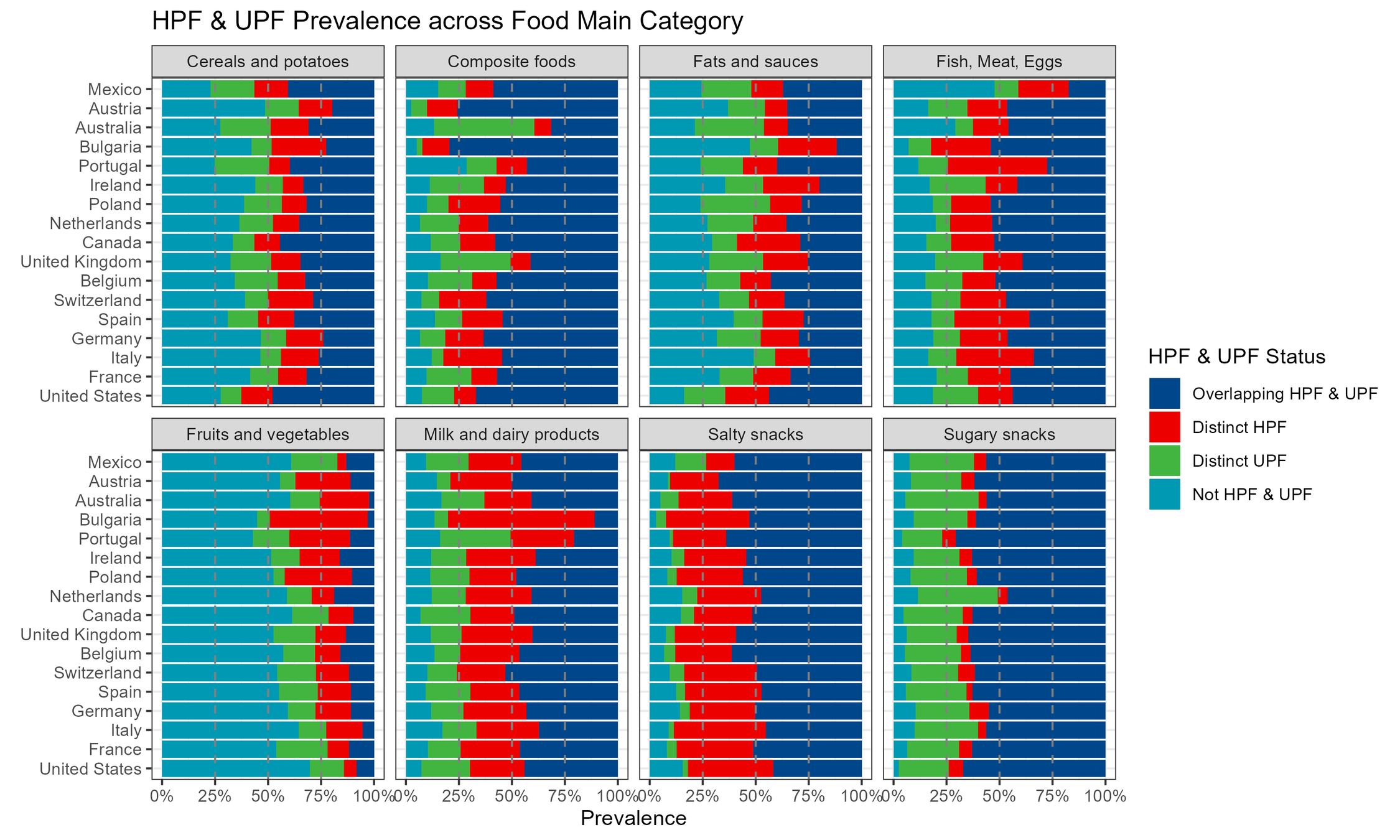

Supplement: S2 File — S1 Fig. Data Source Composition by Country. S2 Fig. The Proportion of Main Food Category within Each Sampled Country. S3 Fig. Prevalence of HPF groups within Food Main Categories across Countries. S4 Fig. 95% Confidence Interval for Odds Ratio of food items being FSOD compared to United States. S5 Fig. 95% Confidence Interval for Odds Ratio of food items being FS compared to United States.S6 Fig. 95% Confidence Interval for Odds Ratio of food items being CSOD compared to United States.S7 Fig. Boxplot for the nutritional compositions of FSOD across countries.S8 Fig. Boxplot for the nutritional compositions of FS across countries.S9 Fig. Boxplot for the nutritional compositions of CSOD across countries. S10 Fig. 95% Confidence intervals plot for nutritional compositions of FSOD compared to the United States.S11 Fig. Confidence intervals plot for nutritional compositions of FS compared to the United States.S12 Fig. Confidence intervals plot for nutritional compositions of CSOD compared to the United States. S13 Fig. Distinct and overlapping prevalence between HPF and UPF across countries within food main categories. (ZIP) [file pone.0325479.s002.zip › S13 Fig.tif]

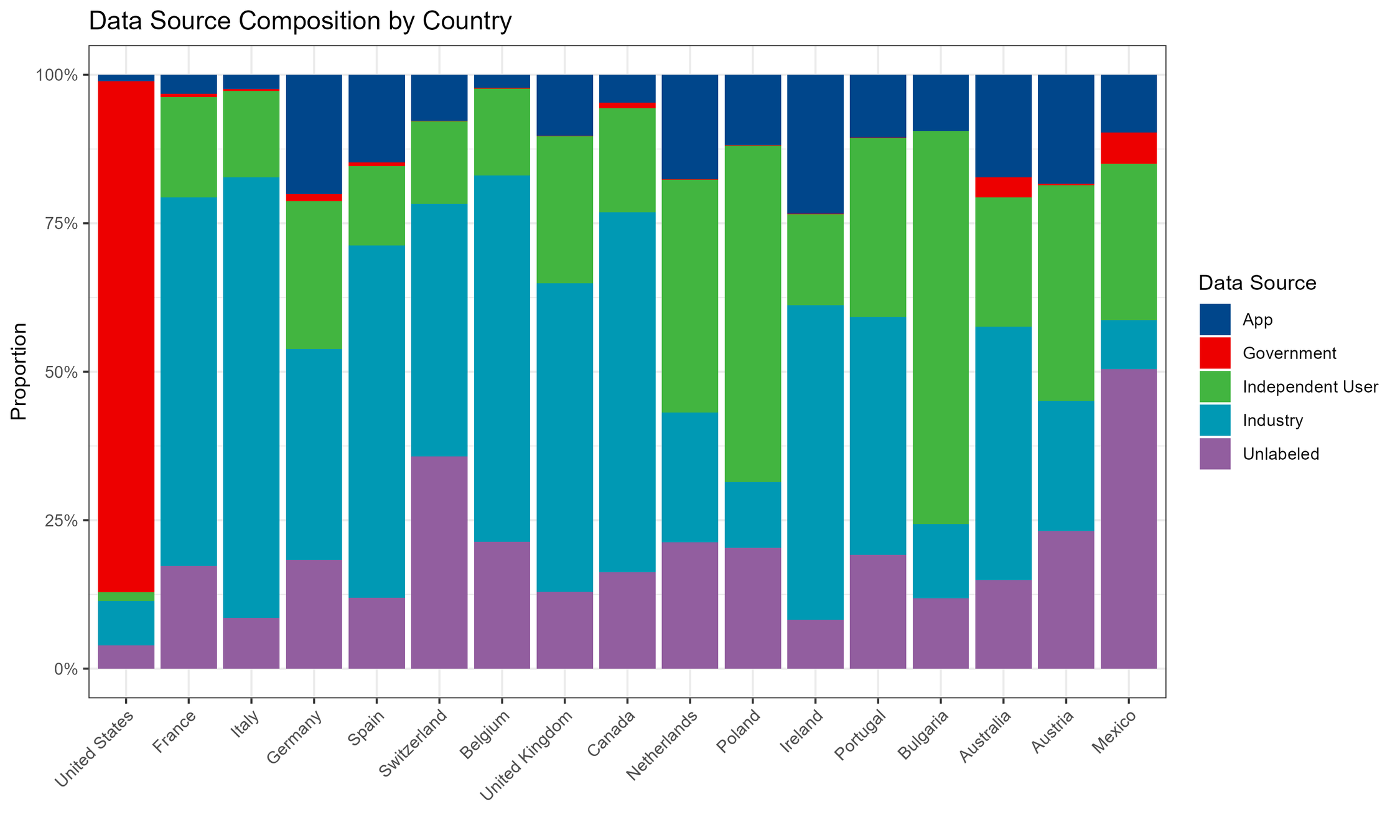

Supplement: S2 File — S1 Fig. Data Source Composition by Country. S2 Fig. The Proportion of Main Food Category within Each Sampled Country. S3 Fig. Prevalence of HPF groups within Food Main Categories across Countries. S4 Fig. 95% Confidence Interval for Odds Ratio of food items being FSOD compared to United States. S5 Fig. 95% Confidence Interval for Odds Ratio of food items being FS compared to United States.S6 Fig. 95% Confidence Interval for Odds Ratio of food items being CSOD compared to United States.S7 Fig. Boxplot for the nutritional compositions of FSOD across countries.S8 Fig. Boxplot for the nutritional compositions of FS across countries.S9 Fig. Boxplot for the nutritional compositions of CSOD across countries. S10 Fig. 95% Confidence intervals plot for nutritional compositions of FSOD compared to the United States.S11 Fig. Confidence intervals plot for nutritional compositions of FS compared to the United States.S12 Fig. Confidence intervals plot for nutritional compositions of CSOD compared to the United States. S13 Fig. Distinct and overlapping prevalence between HPF and UPF across countries within food main categories. (ZIP) [file pone.0325479.s002.zip › S1 Fig.tif]

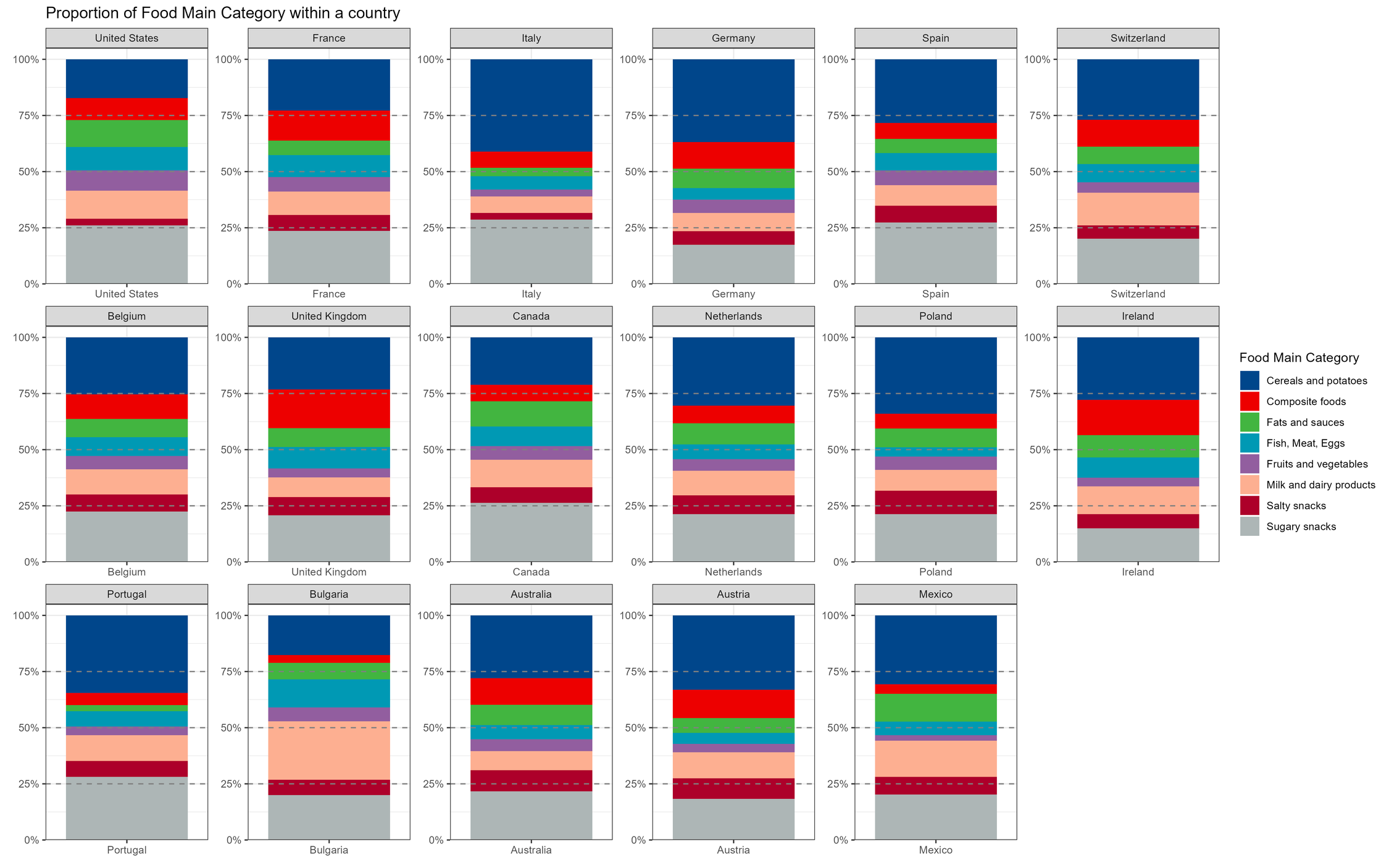

Supplement: S2 File — S1 Fig. Data Source Composition by Country. S2 Fig. The Proportion of Main Food Category within Each Sampled Country. S3 Fig. Prevalence of HPF groups within Food Main Categories across Countries. S4 Fig. 95% Confidence Interval for Odds Ratio of food items being FSOD compared to United States. S5 Fig. 95% Confidence Interval for Odds Ratio of food items being FS compared to United States.S6 Fig. 95% Confidence Interval for Odds Ratio of food items being CSOD compared to United States.S7 Fig. Boxplot for the nutritional compositions of FSOD across countries.S8 Fig. Boxplot for the nutritional compositions of FS across countries.S9 Fig. Boxplot for the nutritional compositions of CSOD across countries. S10 Fig. 95% Confidence intervals plot for nutritional compositions of FSOD compared to the United States.S11 Fig. Confidence intervals plot for nutritional compositions of FS compared to the United States.S12 Fig. Confidence intervals plot for nutritional compositions of CSOD compared to the United States. S13 Fig. Distinct and overlapping prevalence between HPF and UPF across countries within food main categories. (ZIP) [file pone.0325479.s002.zip › S2 Fig.tif]

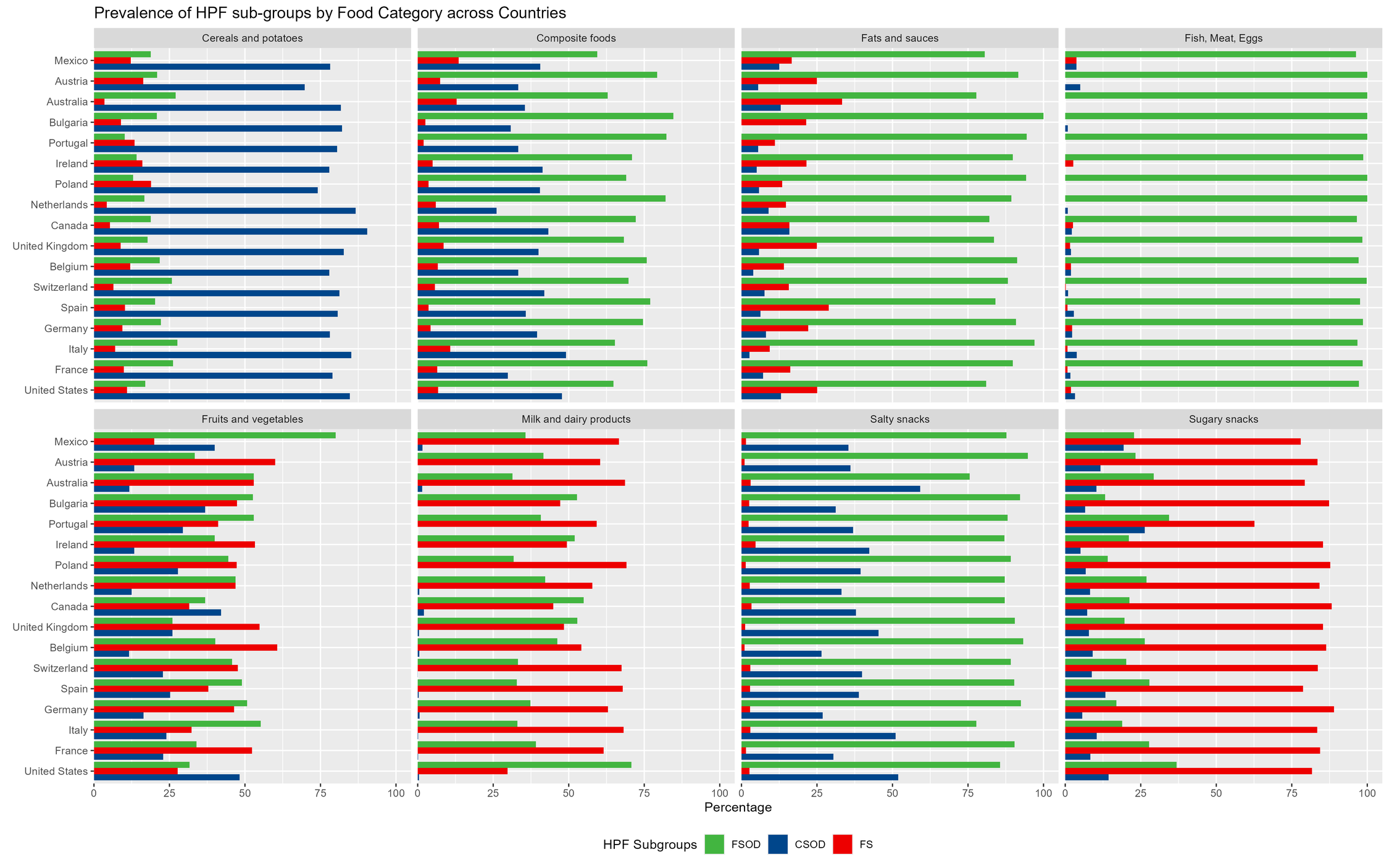

Supplement: S2 File — S1 Fig. Data Source Composition by Country. S2 Fig. The Proportion of Main Food Category within Each Sampled Country. S3 Fig. Prevalence of HPF groups within Food Main Categories across Countries. S4 Fig. 95% Confidence Interval for Odds Ratio of food items being FSOD compared to United States. S5 Fig. 95% Confidence Interval for Odds Ratio of food items being FS compared to United States.S6 Fig. 95% Confidence Interval for Odds Ratio of food items being CSOD compared to United States.S7 Fig. Boxplot for the nutritional compositions of FSOD across countries.S8 Fig. Boxplot for the nutritional compositions of FS across countries.S9 Fig. Boxplot for the nutritional compositions of CSOD across countries. S10 Fig. 95% Confidence intervals plot for nutritional compositions of FSOD compared to the United States.S11 Fig. Confidence intervals plot for nutritional compositions of FS compared to the United States.S12 Fig. Confidence intervals plot for nutritional compositions of CSOD compared to the United States. S13 Fig. Distinct and overlapping prevalence between HPF and UPF across countries within food main categories. (ZIP) [file pone.0325479.s002.zip › S3 Fig.tif]

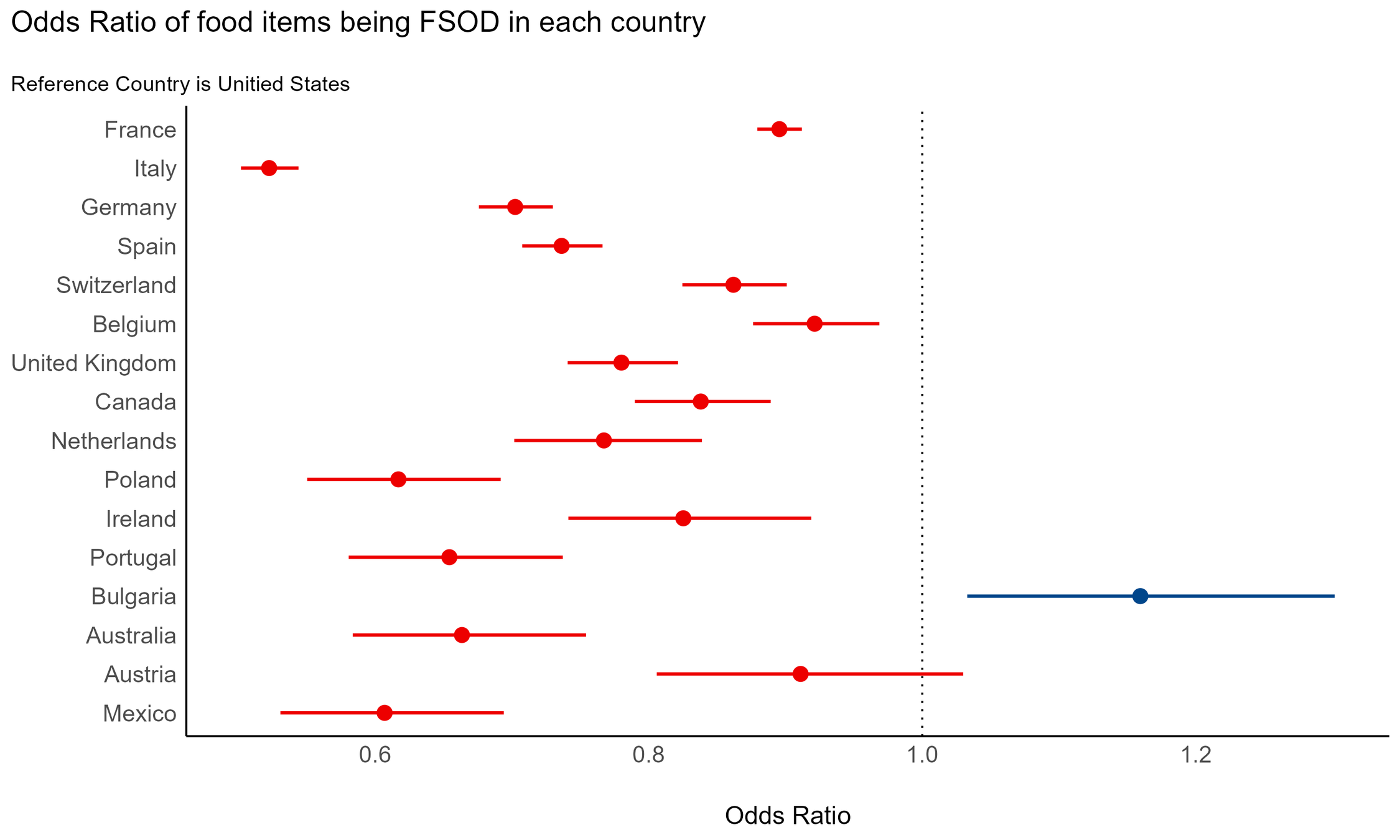

Supplement: S2 File — S1 Fig. Data Source Composition by Country. S2 Fig. The Proportion of Main Food Category within Each Sampled Country. S3 Fig. Prevalence of HPF groups within Food Main Categories across Countries. S4 Fig. 95% Confidence Interval for Odds Ratio of food items being FSOD compared to United States. S5 Fig. 95% Confidence Interval for Odds Ratio of food items being FS compared to United States.S6 Fig. 95% Confidence Interval for Odds Ratio of food items being CSOD compared to United States.S7 Fig. Boxplot for the nutritional compositions of FSOD across countries.S8 Fig. Boxplot for the nutritional compositions of FS across countries.S9 Fig. Boxplot for the nutritional compositions of CSOD across countries. S10 Fig. 95% Confidence intervals plot for nutritional compositions of FSOD compared to the United States.S11 Fig. Confidence intervals plot for nutritional compositions of FS compared to the United States.S12 Fig. Confidence intervals plot for nutritional compositions of CSOD compared to the United States. S13 Fig. Distinct and overlapping prevalence between HPF and UPF across countries within food main categories. (ZIP) [file pone.0325479.s002.zip › S4 Fig.tif]

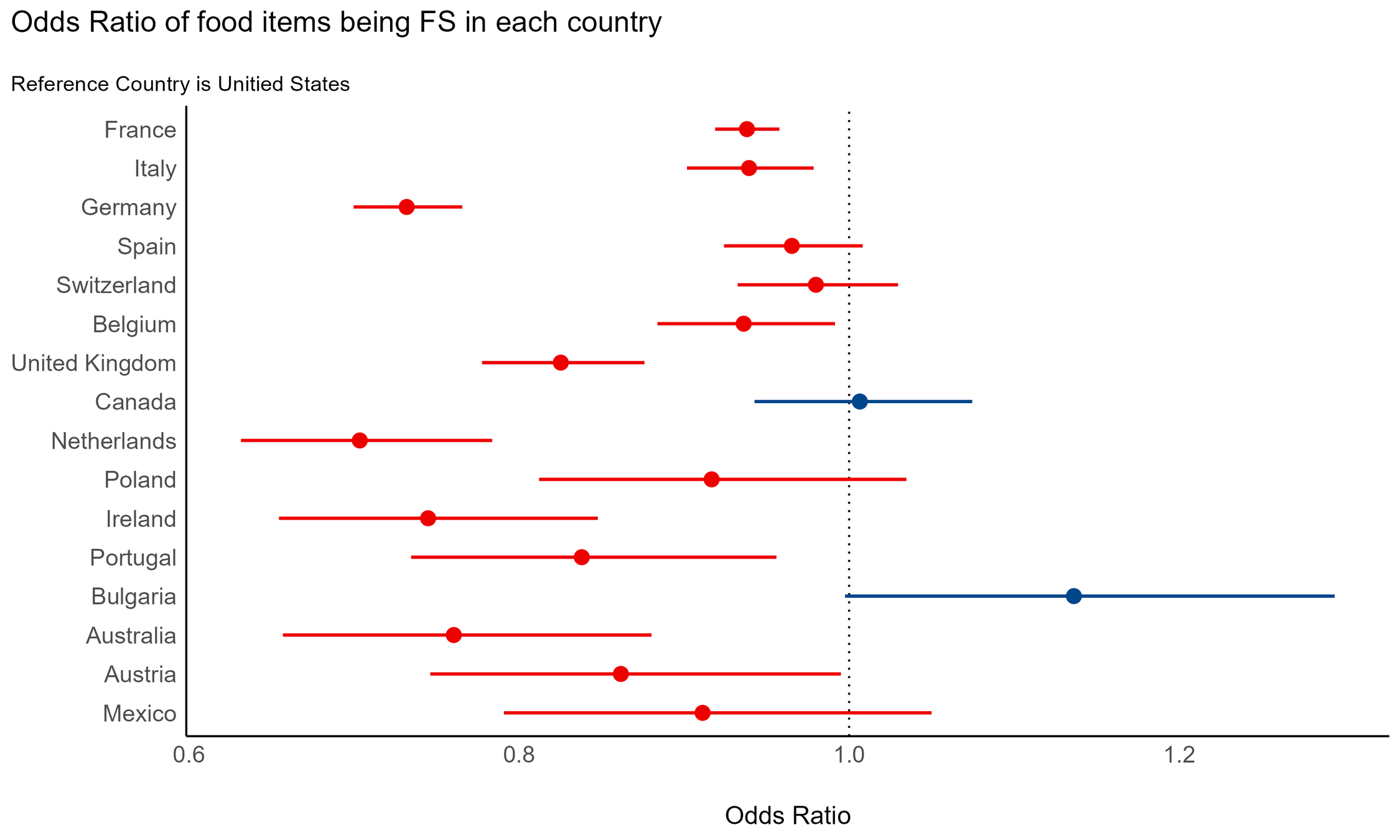

Supplement: S2 File — S1 Fig. Data Source Composition by Country. S2 Fig. The Proportion of Main Food Category within Each Sampled Country. S3 Fig. Prevalence of HPF groups within Food Main Categories across Countries. S4 Fig. 95% Confidence Interval for Odds Ratio of food items being FSOD compared to United States. S5 Fig. 95% Confidence Interval for Odds Ratio of food items being FS compared to United States.S6 Fig. 95% Confidence Interval for Odds Ratio of food items being CSOD compared to United States.S7 Fig. Boxplot for the nutritional compositions of FSOD across countries.S8 Fig. Boxplot for the nutritional compositions of FS across countries.S9 Fig. Boxplot for the nutritional compositions of CSOD across countries. S10 Fig. 95% Confidence intervals plot for nutritional compositions of FSOD compared to the United States.S11 Fig. Confidence intervals plot for nutritional compositions of FS compared to the United States.S12 Fig. Confidence intervals plot for nutritional compositions of CSOD compared to the United States. S13 Fig. Distinct and overlapping prevalence between HPF and UPF across countries within food main categories. (ZIP) [file pone.0325479.s002.zip › S5 Fig.tif]

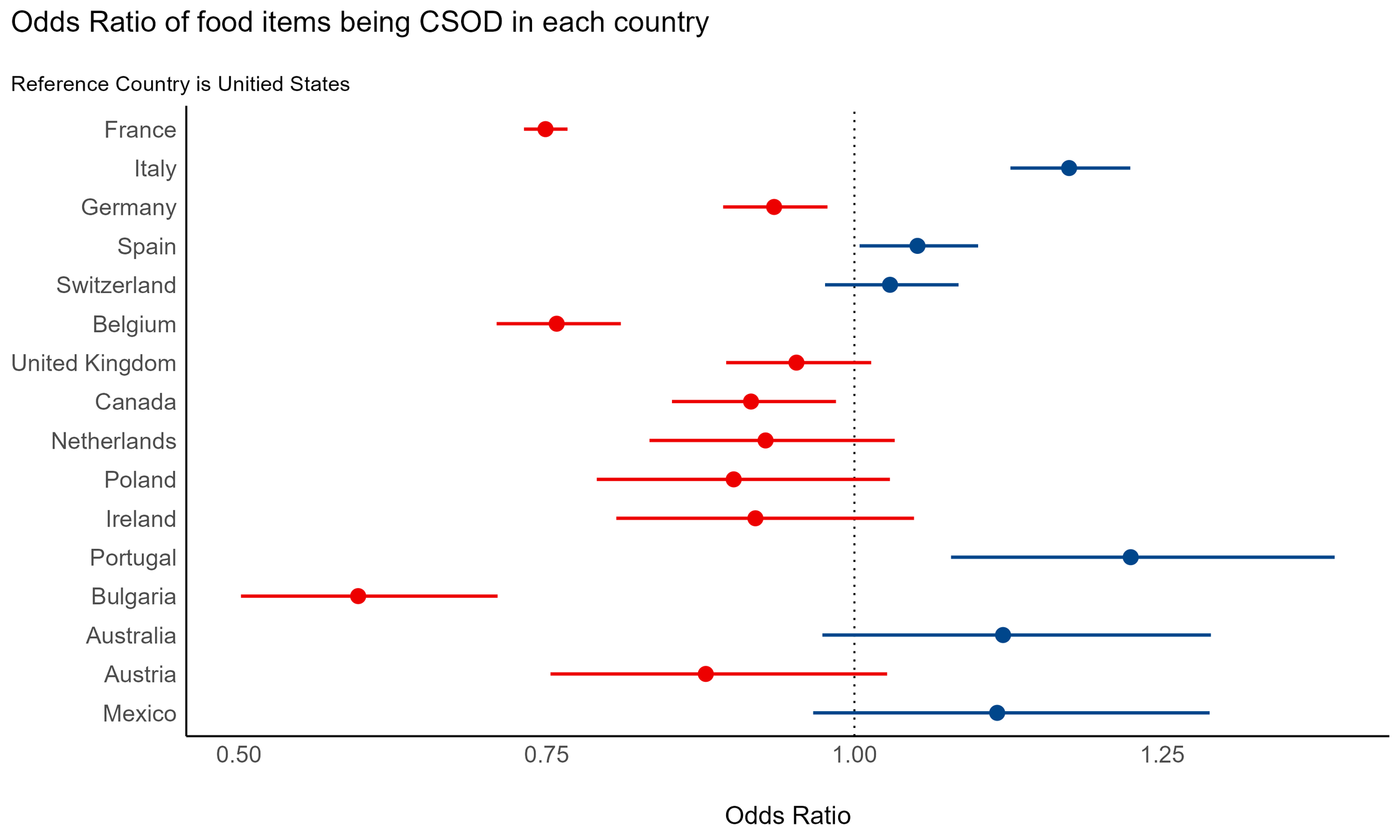

Supplement: S2 File — S1 Fig. Data Source Composition by Country. S2 Fig. The Proportion of Main Food Category within Each Sampled Country. S3 Fig. Prevalence of HPF groups within Food Main Categories across Countries. S4 Fig. 95% Confidence Interval for Odds Ratio of food items being FSOD compared to United States. S5 Fig. 95% Confidence Interval for Odds Ratio of food items being FS compared to United States.S6 Fig. 95% Confidence Interval for Odds Ratio of food items being CSOD compared to United States.S7 Fig. Boxplot for the nutritional compositions of FSOD across countries.S8 Fig. Boxplot for the nutritional compositions of FS across countries.S9 Fig. Boxplot for the nutritional compositions of CSOD across countries. S10 Fig. 95% Confidence intervals plot for nutritional compositions of FSOD compared to the United States.S11 Fig. Confidence intervals plot for nutritional compositions of FS compared to the United States.S12 Fig. Confidence intervals plot for nutritional compositions of CSOD compared to the United States. S13 Fig. Distinct and overlapping prevalence between HPF and UPF across countries within food main categories. (ZIP) [file pone.0325479.s002.zip › S6 Fig.tif]

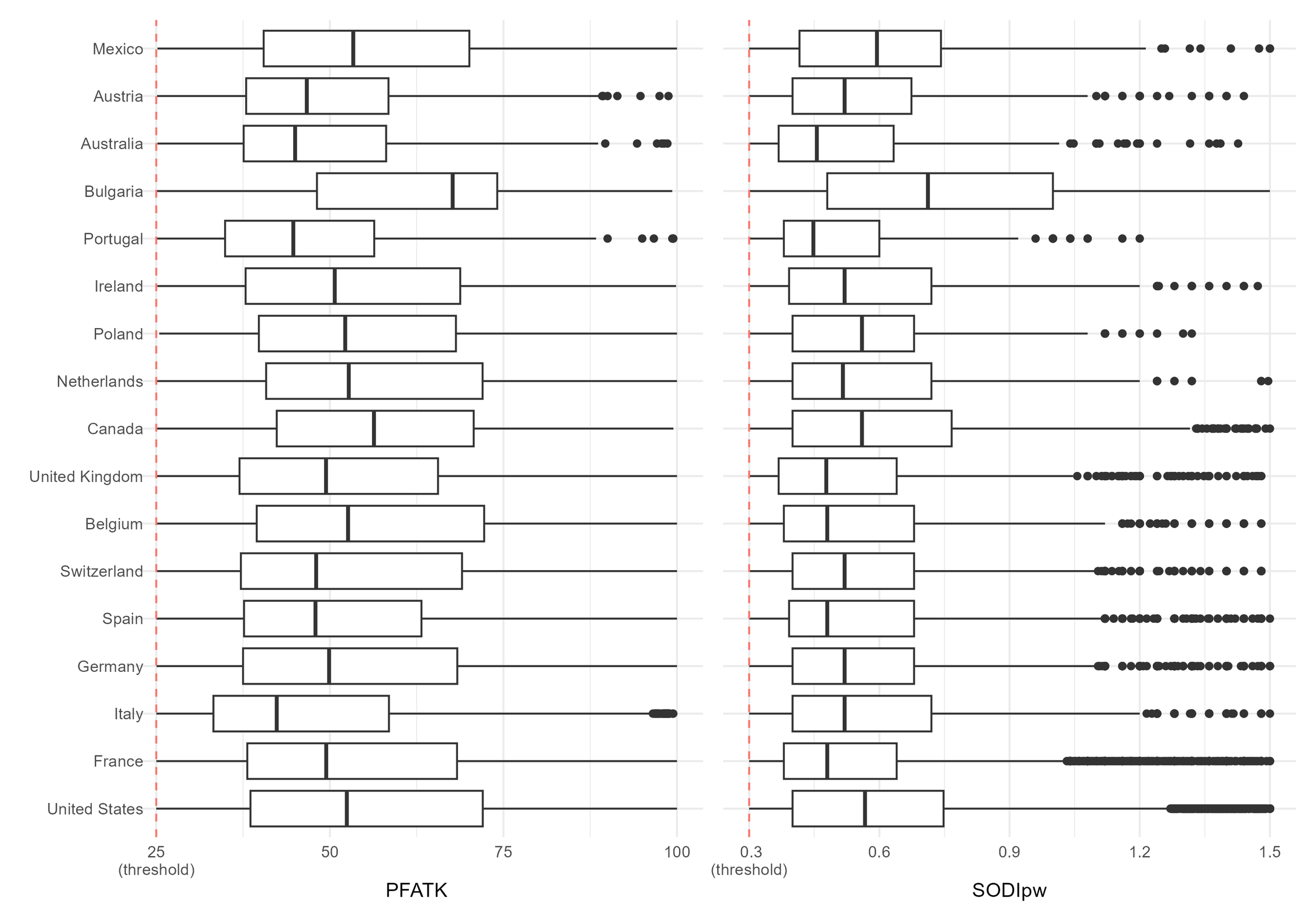

Supplement: S2 File — S1 Fig. Data Source Composition by Country. S2 Fig. The Proportion of Main Food Category within Each Sampled Country. S3 Fig. Prevalence of HPF groups within Food Main Categories across Countries. S4 Fig. 95% Confidence Interval for Odds Ratio of food items being FSOD compared to United States. S5 Fig. 95% Confidence Interval for Odds Ratio of food items being FS compared to United States.S6 Fig. 95% Confidence Interval for Odds Ratio of food items being CSOD compared to United States.S7 Fig. Boxplot for the nutritional compositions of FSOD across countries.S8 Fig. Boxplot for the nutritional compositions of FS across countries.S9 Fig. Boxplot for the nutritional compositions of CSOD across countries. S10 Fig. 95% Confidence intervals plot for nutritional compositions of FSOD compared to the United States.S11 Fig. Confidence intervals plot for nutritional compositions of FS compared to the United States.S12 Fig. Confidence intervals plot for nutritional compositions of CSOD compared to the United States. S13 Fig. Distinct and overlapping prevalence between HPF and UPF across countries within food main categories. (ZIP) [file pone.0325479.s002.zip › S7 Fig.tif]

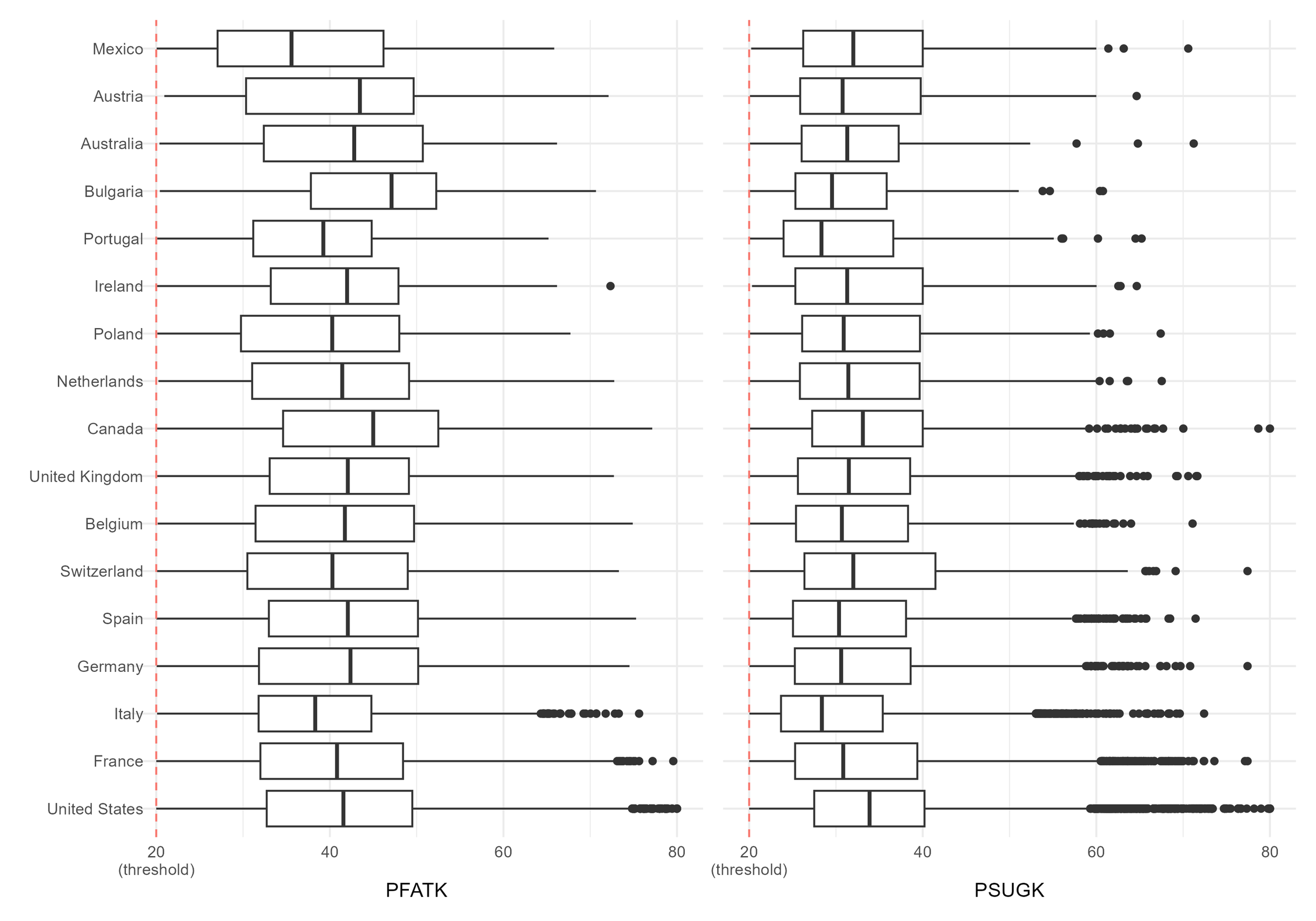

Supplement: S2 File — S1 Fig. Data Source Composition by Country. S2 Fig. The Proportion of Main Food Category within Each Sampled Country. S3 Fig. Prevalence of HPF groups within Food Main Categories across Countries. S4 Fig. 95% Confidence Interval for Odds Ratio of food items being FSOD compared to United States. S5 Fig. 95% Confidence Interval for Odds Ratio of food items being FS compared to United States.S6 Fig. 95% Confidence Interval for Odds Ratio of food items being CSOD compared to United States.S7 Fig. Boxplot for the nutritional compositions of FSOD across countries.S8 Fig. Boxplot for the nutritional compositions of FS across countries.S9 Fig. Boxplot for the nutritional compositions of CSOD across countries. S10 Fig. 95% Confidence intervals plot for nutritional compositions of FSOD compared to the United States.S11 Fig. Confidence intervals plot for nutritional compositions of FS compared to the United States.S12 Fig. Confidence intervals plot for nutritional compositions of CSOD compared to the United States. S13 Fig. Distinct and overlapping prevalence between HPF and UPF across countries within food main categories. (ZIP) [file pone.0325479.s002.zip › S8 Fig.tif]

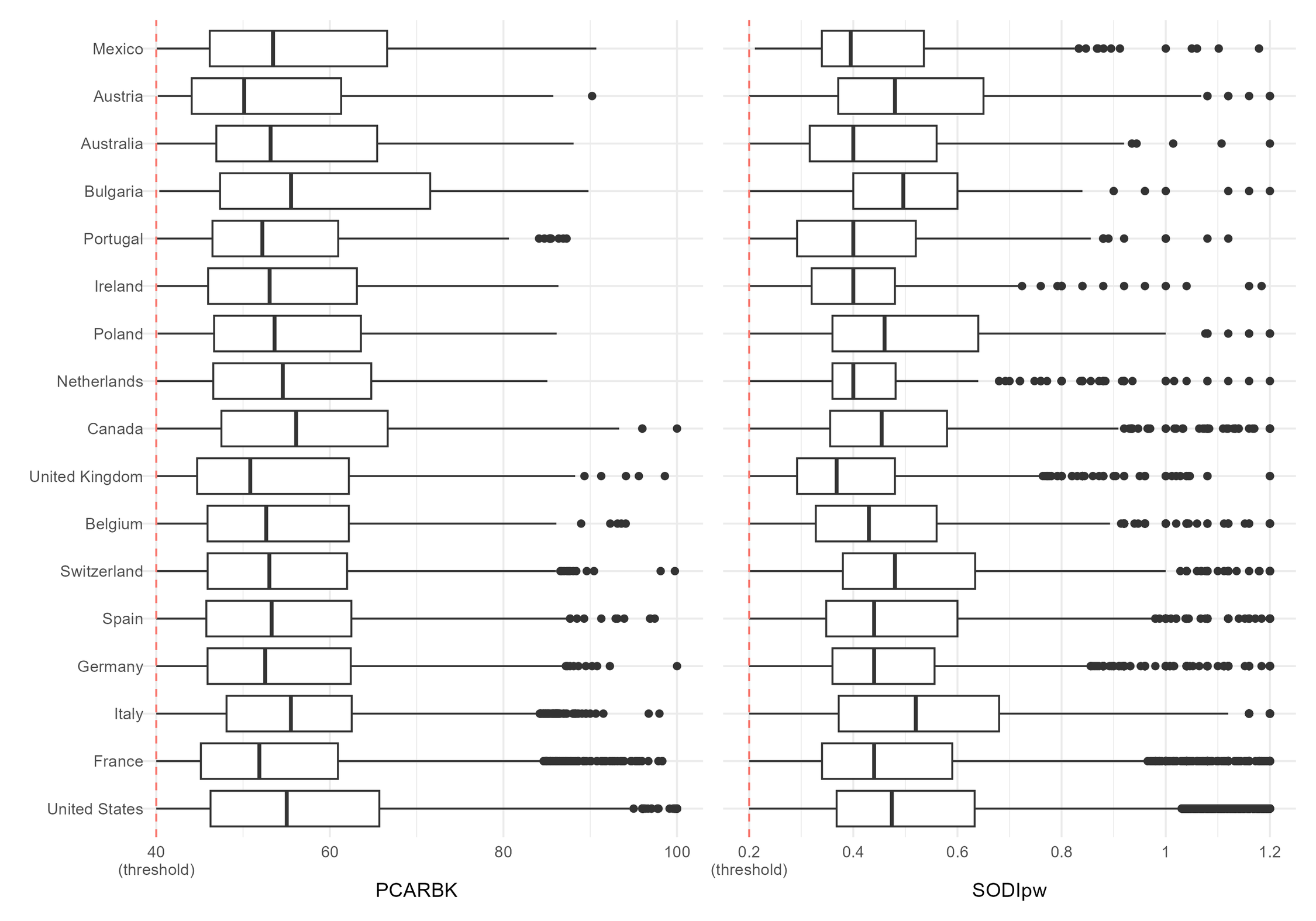

Supplement: S2 File — S1 Fig. Data Source Composition by Country. S2 Fig. The Proportion of Main Food Category within Each Sampled Country. S3 Fig. Prevalence of HPF groups within Food Main Categories across Countries. S4 Fig. 95% Confidence Interval for Odds Ratio of food items being FSOD compared to United States. S5 Fig. 95% Confidence Interval for Odds Ratio of food items being FS compared to United States.S6 Fig. 95% Confidence Interval for Odds Ratio of food items being CSOD compared to United States.S7 Fig. Boxplot for the nutritional compositions of FSOD across countries.S8 Fig. Boxplot for the nutritional compositions of FS across countries.S9 Fig. Boxplot for the nutritional compositions of CSOD across countries. S10 Fig. 95% Confidence intervals plot for nutritional compositions of FSOD compared to the United States.S11 Fig. Confidence intervals plot for nutritional compositions of FS compared to the United States.S12 Fig. Confidence intervals plot for nutritional compositions of CSOD compared to the United States. S13 Fig. Distinct and overlapping prevalence between HPF and UPF across countries within food main categories. (ZIP) [file pone.0325479.s002.zip › S9 Fig.tif]

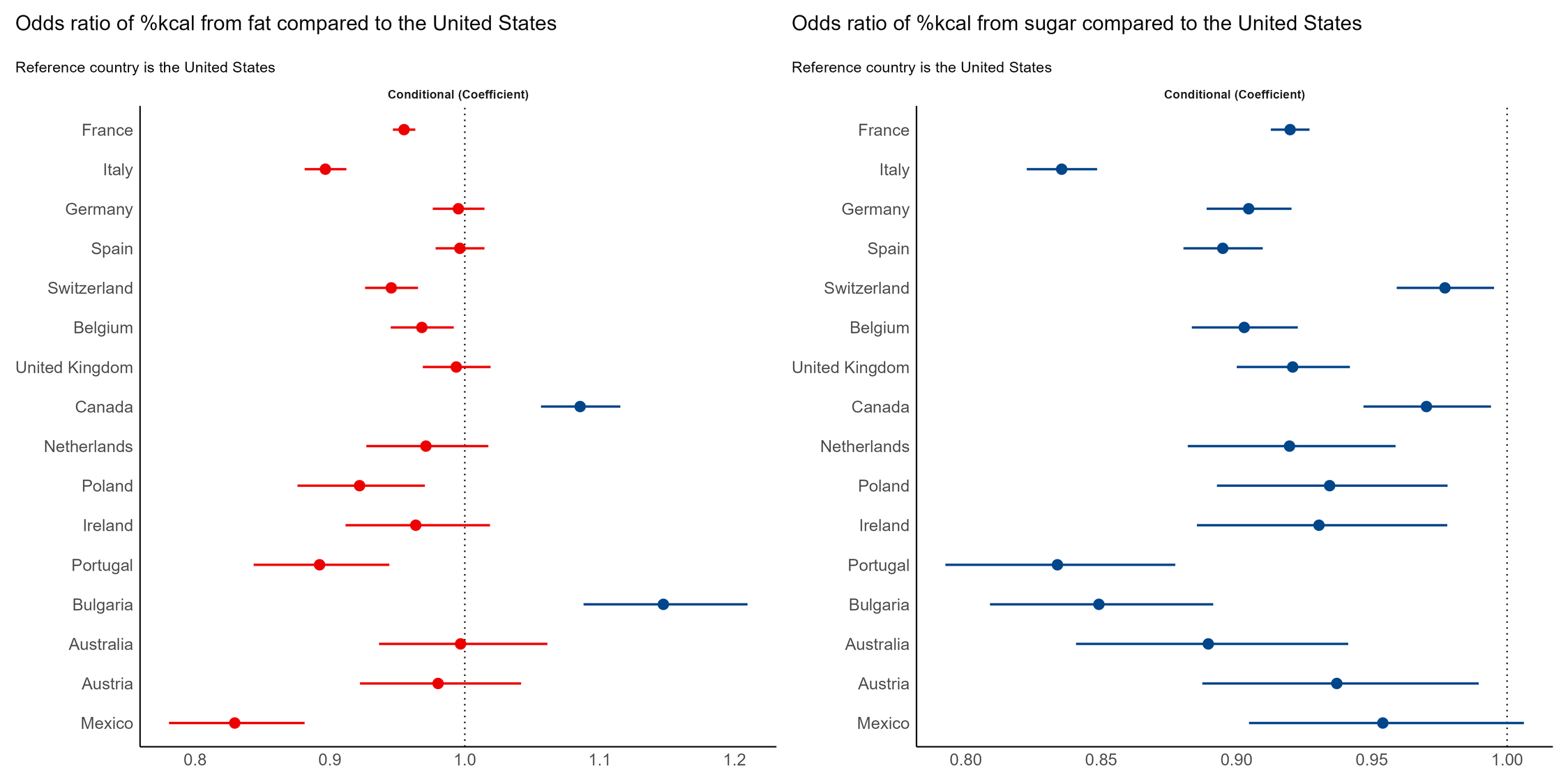

Supplement: S2 File — S1 Fig. Data Source Composition by Country. S2 Fig. The Proportion of Main Food Category within Each Sampled Country. S3 Fig. Prevalence of HPF groups within Food Main Categories across Countries. S4 Fig. 95% Confidence Interval for Odds Ratio of food items being FSOD compared to United States. S5 Fig. 95% Confidence Interval for Odds Ratio of food items being FS compared to United States.S6 Fig. 95% Confidence Interval for Odds Ratio of food items being CSOD compared to United States.S7 Fig. Boxplot for the nutritional compositions of FSOD across countries.S8 Fig. Boxplot for the nutritional compositions of FS across countries.S9 Fig. Boxplot for the nutritional compositions of CSOD across countries. S10 Fig. 95% Confidence intervals plot for nutritional compositions of FSOD compared to the United States.S11 Fig. Confidence intervals plot for nutritional compositions of FS compared to the United States.S12 Fig. Confidence intervals plot for nutritional compositions of CSOD compared to the United States. S13 Fig. Distinct and overlapping prevalence between HPF and UPF across countries within food main categories. (ZIP) [file pone.0325479.s002.zip › S10 Fig.tif]

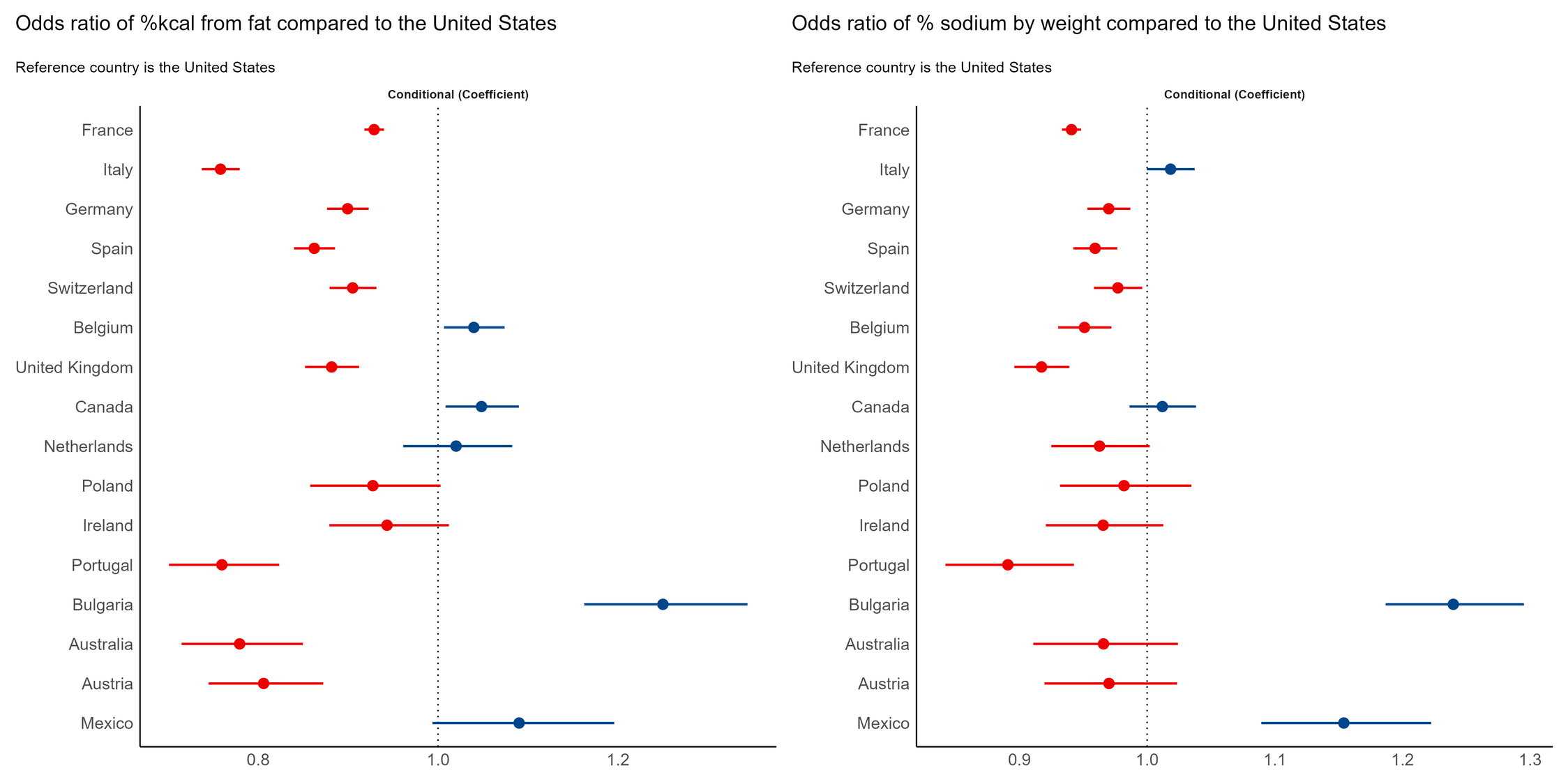

Supplement: S2 File — S1 Fig. Data Source Composition by Country. S2 Fig. The Proportion of Main Food Category within Each Sampled Country. S3 Fig. Prevalence of HPF groups within Food Main Categories across Countries. S4 Fig. 95% Confidence Interval for Odds Ratio of food items being FSOD compared to United States. S5 Fig. 95% Confidence Interval for Odds Ratio of food items being FS compared to United States.S6 Fig. 95% Confidence Interval for Odds Ratio of food items being CSOD compared to United States.S7 Fig. Boxplot for the nutritional compositions of FSOD across countries.S8 Fig. Boxplot for the nutritional compositions of FS across countries.S9 Fig. Boxplot for the nutritional compositions of CSOD across countries. S10 Fig. 95% Confidence intervals plot for nutritional compositions of FSOD compared to the United States.S11 Fig. Confidence intervals plot for nutritional compositions of FS compared to the United States.S12 Fig. Confidence intervals plot for nutritional compositions of CSOD compared to the United States. S13 Fig. Distinct and overlapping prevalence between HPF and UPF across countries within food main categories. (ZIP) [file pone.0325479.s002.zip › S11 Fig.tif]

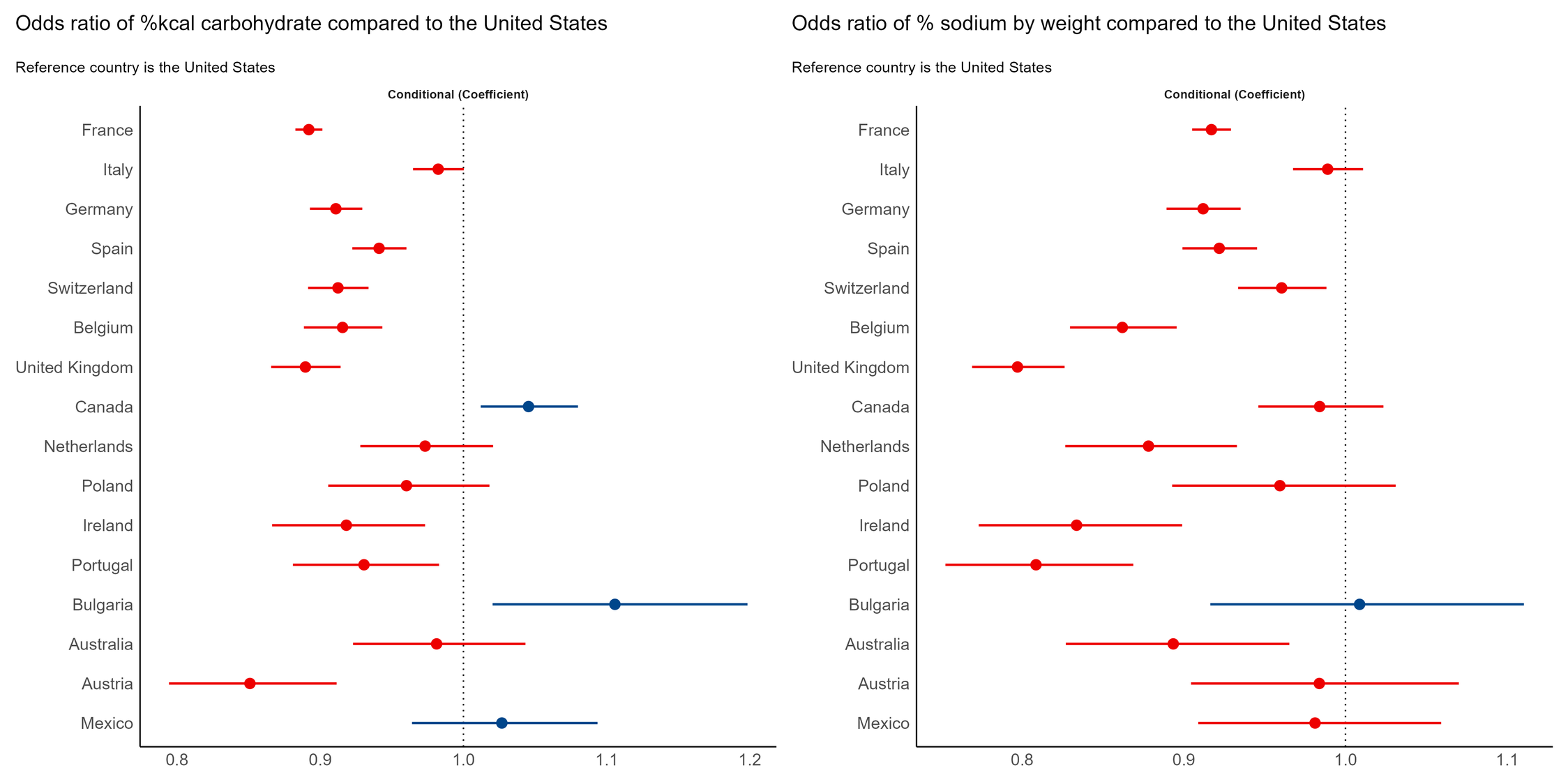

Supplement: S2 File — S1 Fig. Data Source Composition by Country. S2 Fig. The Proportion of Main Food Category within Each Sampled Country. S3 Fig. Prevalence of HPF groups within Food Main Categories across Countries. S4 Fig. 95% Confidence Interval for Odds Ratio of food items being FSOD compared to United States. S5 Fig. 95% Confidence Interval for Odds Ratio of food items being FS compared to United States.S6 Fig. 95% Confidence Interval for Odds Ratio of food items being CSOD compared to United States.S7 Fig. Boxplot for the nutritional compositions of FSOD across countries.S8 Fig. Boxplot for the nutritional compositions of FS across countries.S9 Fig. Boxplot for the nutritional compositions of CSOD across countries. S10 Fig. 95% Confidence intervals plot for nutritional compositions of FSOD compared to the United States.S11 Fig. Confidence intervals plot for nutritional compositions of FS compared to the United States.S12 Fig. Confidence intervals plot for nutritional compositions of CSOD compared to the United States. S13 Fig. Distinct and overlapping prevalence between HPF and UPF across countries within food main categories. (ZIP) [file pone.0325479.s002.zip › S12 Fig.tif]
