# Supplementary material for: Examination of hyper-palatable foods and their nutrient characteristics using globally crowdsourced data
Source: PLoS One. 2025 Jun 6;20(6):e0325479. doi: 10.1371/journal.pone.0325479 (PMC12143524; doi:10.1371/journal.pone.0325479)
Supplement: Attachment — Submitted filename: Review_PLOS_ONE_HPF_Structure.pdf [file pone.0325479.s004.pdf]

Uberlândia, 16 de April de 2025

To the Editors,

PLOS ONE

Dear Editors,

Thank you for the opportunity to serve as a reviewer for the manuscript entitled "Examination of Hyper-Palatable Foods and their Nutrient Characteristics using Globally Crowdsourced Data" (Manuscript ID: PONE-D-24-53803R1). I am grateful for the chance to contribute to the evaluation of such an important and timely piece of work.

This is a well-structured manuscript that addresses an emerging issue of global relevance — the prevalence and nutritional characteristics of hyper-palatable foods (HPF, hyper-palatable foods) in comparison to ultra-processed foods (UPF, ultra-processed foods), using a globally crowdsourced dataset. The study employs an impressive dataset and applies appropriate statistical methods to explore international variations, contributing to a growing body of literature on food environments and public health.

While the manuscript presents a compelling and methodologically sound analysis, and the authors have taken care to acknowledge its inherent limitations, I offer below a number of observations and suggestions that may help further improve the clarity, rigor, and interpretability of the work.

Sincerely,

Sinara Laurini Rossato
Adjunct Professor at Universidade Federal de Uberlândia

1. Summary of the Manuscript

The manuscript titled "Examination of Hyper-Palatable Foods and their Nutrient Characteristics using Globally Crowdsourced Data" presents a well-organized cross-national analysis of over 314,000 food items from 17 countries, using the Open Food Facts database. The authors apply a standardized definition of hyper-palatable foods (HPF, hyper-palatable foods) and compare their prevalence, nutrient composition, and overlap with ultra-processed foods (UPF, ultra-processed foods) based on the NOVA classification. The findings show notably higher HPF prevalence and nutrient excess in the United States, while countries like Italy and Germany show lower levels. The study offers a relevant and methodologically sound contribution to food system analysis and public health nutrition.

2. Assessment of Methodological Rigor

The manuscript titled "Examination of Hyper-Palatable Foods and their Nutrient Characteristics using Globally Crowdsourced Data" presents a cross-national analysis of hyper-palatable foods (HPF, hyper-palatable foods) using over 314,000 food items from the Open Food Facts (OFF) database, spanning 17 countries da Europa, América do Norte e América do Sul. HPF were identified through a validated, nutrient-based definition that captures combinations such as fat and sodium, fat and sugar, or carbohydrate and sodium [1].

The study compares the prevalence and nutrient profiles of HPF across countries and analyzes their overlap with ultra-processed foods (UPF, ultra-processed foods), as defined by the NOVA classification [2]. Results show that food items from the United States are significantly more likely to be classified as HPF and tend to exceed threshold criteria by larger margins, whereas countries like Italy and Germany present notably lower prevalence and intensity of HPF profiles [3].

This work is methodologically transparent, statistically sound, and provides a novel contribution to global food environment research. It highlights important distinctions between HPF and UPF and offers insights relevant to regulatory strategies, especially in high-saturation food systems such as those in the United States.

To further strengthen the methodological transparency and interpretability of the study, I would recommend that the authors provide additional context regarding the representativeness and potential biases of the dataset. Specifically, a more detailed discussion of how national differences in data entry patterns, contributor types (e.g.,

individual users, automated imports, industry uploads), or data completeness may have affected the comparability across countries would be valuable.

In addition, although formal population-based weighting is not feasible within the Open Food Facts (OFF) platform, I would encourage the authors to explore the possibility of incorporating a basic coverage index per country (e.g., items per capita, items per known market share category) to provide a clearer sense of how over- or underrepresented each country's food supply may be within the dataset. Even a descriptive indicator of coverage disparity would aid in contextualizing the results.

Lastly, I suggest that future studies building on this work consider triangulating findings with complementary sources of food environment or consumption data (e.g., NHANES, Euromonitor, or national food composition tables) to enhance the external validity of results and support policy-relevant interpretations.

3. Specific Comments

3.1 Title and Abstract

The current title is clear and accurately reflects the scope of the study; however, two small refinements could enhance its precision and accessibility. First, given that the manuscript includes a substantial comparative analysis between hyper-palatable foods (HPF) and ultra-processed foods (UPF), it is recommended to incorporate this dimension explicitly in the title (e.g., "…and Their Overlap with Ultra-Processed Foods"). Second, while the term "crowdsourced data" is technically correct, adding a reference to the Open Food Facts platform in the title (e.g., "…using Data from Open Food Facts") may improve clarity for broader audiences less familiar with crowdsourcing terminology.

The abstract provides a clear and well-structured summary of the study; however, several refinements could enhance its clarity and impact. First, it is recommended to mention the specific data source — Open Food Facts — explicitly, rather than referring only to a "globally crowdsourced, open-source dataset." Naming the platform would improve transparency and reproducibility. Second, because the comparison between hyper-palatable foods (HPF) and ultra-processed foods (UPF) is a major contribution of the manuscript, this element should be introduced earlier in the abstract, ideally in the second sentence. Lastly, the concluding sentence could be strengthened by more clearly articulating the public health relevance or policy implications of the findings, as this is a central point of the Discussion section.

The abstract is grammatically sound and well-organized; however, there are a few stylistic adjustments that could improve clarity and readability. For example, the opening sentence is somewhat dense and could be split into two for greater ease of understanding. Additionally, the phrase “substantially exceeded HPF threshold criteria by 70–229%” is technically correct but might be better phrased more directly, given the precise numeric values already reported. The final sentence of the abstract is somewhat generic and could be strengthened by explicitly referencing public health or policy relevance. Furthermore, a minor typographical error was found in this last sentence — the acronym “HFP” should read “HPF” (hyper-palatable foods). Addressing these elements would enhance the precision and readability of the abstract.

3.2 Introduction

The introduction establishes a sound rationale for studying hyper-palatable foods (HPF, hyper-palatable foods), drawing upon neurobiological, behavioral, and public health perspectives. The conceptual background is appropriately anchored in the literature, and the authors correctly cite foundational works such as the NOVA classification for ultra-processed foods (UPF, ultra-processed foods) [3] and prior international research on HPF in Italy [4]. However, the section could be improved by addressing a few key points.

First, the narrative could benefit from greater focus. The early paragraphs are somewhat repetitive regarding the neurobiological mechanisms of HPF and could be shortened to emphasize more clearly the empirical knowledge gap. The real novelty of this manuscript lies not in describing the mechanisms of HPF, but in characterizing their global availability — a point that deserves greater prominence.

Second, although UPF is introduced midway through the section, the conceptual contrast and empirical relationship between HPF and UPF are core elements of the study and merit earlier inclusion. This would clarify the added value of the comparative approach and align better with the results and discussion.

Third, while the authors reference the only known prior cross-national comparison (Italy vs. U.S.), additional recent studies could further strengthen their case for public health relevance. For example, national data indicate that nearly half of U.S. elementary school lunches contain HPF, with entrées and side dishes most likely to be hyper-palatable [5]. Furthermore, HPF intake — especially carbohydrate-and-sodium (CSOD) combinations — has been longitudinally linked to weight and body fat gain in

young adults over one year [6]. Including such examples would reinforce the importance of understanding HPF exposure across contexts.

The introduction is grammatically correct and conceptually well-developed. However, several stylistic refinements could improve its clarity and flow. Some sentences are overly long and could be restructured for conciseness, particularly in the early paragraphs describing neurobiological mechanisms.

The repeated use of modal verbs such as “may” and phrases like “hypothesized to” tends to weaken the tone and could be varied or replaced for improved assertiveness. Additionally, the frequent use of parenthetical expressions (e.g., e.g., fat and sodium) could be reduced to enhance readability.

The final paragraph outlining the study’s objectives is informative but structurally dense; rephrasing it into a more compact and parallel form would facilitate reader comprehension. These are not grammatical errors per se, but stylistic enhancements that would elevate the manuscript’s overall readability and academic tone.

3.3 Materials and Methods

The methods section provides an adequate overview of the data source, processing procedures, and analytic strategies. The use of the Open Food Facts (OFF) database is well justified, and the authors describe appropriate filtering criteria to ensure data quality. The explicit inclusion of R code and public access to the cleaned dataset reflects good scientific practice and aligns with PLOS ONE’s emphasis on transparency and reproducibility. Nonetheless, a few clarifications and suggestions are warranted.

First, while the authors mention that quality checks were applied (e.g., removal of duplicates and implausible values), the thresholds for these exclusions are not fully specified. Providing concrete examples or criteria (e.g., nutrient ranges considered implausible) would enhance replicability.

Second, the rationale for excluding beverages due to incompatibility with the HPF (hyper-palatable foods) definition is consistent with prior work [1], but might benefit from a brief justification in the text, since beverages can be substantial contributors to caloric intake and dietary palatability in many settings.

Third, the HPF classification is based on the standardized, data-driven thresholds developed by Fazzino et al. [1], which is appropriate. However, the manuscript does not

clearly state whether any sensitivity analyses were conducted to test the robustness of classification across possible variations in nutrient labeling or serving size inconsistencies — a known issue in crowdsourced databases.

Fourth, the authors' decision to include only countries with more than 1,000 unique food items is practical, but it should be acknowledged that this choice may have excluded countries with different food system structures (e.g., from Asia or Africa), thus potentially limiting the generalizability of the findings.

Lastly, the application of logistic and ordered beta regression models is appropriate for the study's aims. The explanation of why beta regression was used for bounded variables (percentages from 0 to 100) is appreciated, though a bit more detail on model assumptions and diagnostics would further support confidence in the results.

The writing in the Methods section is grammatically correct and technically coherent. Nevertheless, several minor stylistic adjustments could improve clarity and enhance the academic tone of the manuscript.

For example, the sentence "The total food items used for the data analysis were 314229 from 17 countries" is grammatically sound but would benefit from a more fluid and conventional construction such as "A total of 314,229 food items from 17 countries were included in the final analysis."

Similarly, the phrase "The rationale for excluding beverages is that the HPF definition does not apply to beverages…" could be rephrased more directly as "Beverages were excluded as the HPF definition is not applicable to liquid items," which eliminates repetition and improves conciseness.

Additionally, the expression "quality evaluation procedures were conducted" may sound vague and could be replaced by the more precise "quality control procedures included the removal of duplicate entries and implausible nutritional values."

Finally, some passive constructions (e.g., "Food items identified as having a potential data quality issue… were excluded") could be clarified by explicitly stating the criteria or thresholds used. Although these are not grammatical errors, these refinements would enhance the manuscript's readability and professional tone.

3.4 Results

The Results section is comprehensive, well-structured, and clearly aligned with the study's objectives. Descriptive and inferential analyses are logically sequenced and supported by well-designed figures and supplemental materials. The use of logistic and ordered beta regression models is appropriate for the data structure, and the decision to stratify results by HPF (hyper-palatable foods) subgroup — fat and sodium (FSOD), fat and sugar (FS), and carbohydrate and sodium (CSOD) — allows for meaningful comparisons across food types and countries.

One strength of this section is the authors' use of concrete numerical results, such as odds ratios and percentage thresholds, which provide clarity and interpretability for the reader. The presentation of how HPF exceed their defining thresholds across countries is especially informative and reinforces the validity of the classification scheme previously established in the literature [1].

However, a few areas warrant attention. First, while the text refers to extensive supplementary figures and tables, not all of them are summarized in the main text. It may help to briefly highlight key findings from these figures, particularly when discussing cross-country distinctions in HPF nutrient composition.

Second, although the study is not aiming to provide representative national estimates (as acknowledged in the Methods and Discussion), the reader would benefit from a brief reminder in this section that comparisons are exploratory and not population-weighted.

Additionally, the analysis of HPF–UPF overlap is one of the most novel contributions of the manuscript, yet it is somewhat buried toward the end of the section. This could be emphasized earlier and linked back to recent findings from the U.S. food system, which showed a growing overlap of HPF and UPF over the last three decades.

The Results section is written in clear and generally precise language. However, a few minor refinements could improve fluency and strengthen scientific tone. Some phrases are overly mechanical in structure (e.g., repeated use of "items from [country] had significantly lower…"), which could be varied to maintain reader engagement.

Additionally, when describing statistical comparisons, more interpretive language (e.g., "substantially higher," "marginally lower") could be introduced alongside numerical outputs to help contextualize findings.

Finally, transitions between paragraphs or subsections could be enhanced to improve flow, particularly when shifting from descriptive to inferential findings or when introducing the HPF–UPF overlap analysis.

While not grammatical errors per se, these adjustments would enhance narrative cohesion and readability.

3.5 Discussion and Conclusions

The Discussion section concisely synthesizes the main findings and situates them well within the broader literature on food systems, hyper-palatable foods (HPF), and ultra-processed foods (UPF). A key strength lies in the interpretation of HPF subtypes (FSOD, FS, CSOD) and their distribution across food categories, which the authors contextualize through cultural and structural differences in national food environments. Notably, they highlight the higher prevalence of HPF and UPF in the U.S. and the lower presence of such foods in countries like Italy, consistent with earlier studies [3, 4].

Additionally, while the manuscript addresses both HPF and UPF, the conceptual distinction between these constructs — one defined by nutrient-based palatability thresholds and the other by degree and purpose of industrial processing — could be clarified further in the introduction or discussion. Although this relationship is likely familiar to researchers in this field, making it more explicit would benefit readers and enhance the interpretability of the comparative findings.

That said, a few areas could be improved. First, while the discussion of HPF–UPF overlap is well-developed, the authors might better articulate the conceptual distinction between the two — specifically, that UPF classification is based on processing and ingredients, whereas HPF relates directly to nutrient-driven palatability and reinforcement mechanisms [2]. Making this distinction more explicit would enhance the reader's understanding of why examining both constructs simultaneously is valuable.

Second, although the authors briefly mention health implications, this section could more clearly differentiate between epidemiological evidence of HPF's association with health outcomes (e.g., increased adiposity) [6] and concerns linked specifically to UPF (e.g., metabolic and inflammatory pathways) [7]. Doing so would reinforce the manuscript's conclusion that these classifications capture overlapping but non-redundant health risks.

Finally, the proposed public health measures (e.g., reformulation, product repositioning, marketing restrictions) are thought-provoking and aligned with the evidence presented. However, they might benefit from additional nuance — for instance, acknowledging the complexity of regulating HPF in the absence of a universally adopted legal or nutritional standard.

The Discussion and Conclusions section is clearly written and grammatically sound. The authors demonstrate fluency in academic writing, though minor stylistic refinements could improve flow. Several paragraphs begin with factual restatements and would benefit from more interpretive framing. The final paragraph could also use clearer transitions and a sharper distinction between short- and long-term recommendations.

3.6 Figures and Tables

Figures and tables are well-designed and effectively support the study's findings, especially in visualizing HPF and UPF comparisons. The stacked bar chart showing HPF/UPF overlap adds interpretive value. While supplementary materials are extensive, synthesizing key trends within the main text—especially cross-country contrasts—would improve clarity. Most figures are well-labeled, but some legends could offer more interpretive guidance, and axis labels in supplemental plots could be larger. Overall, presentation is solid but would benefit from minor refinements for better integration and readability.

3.7 References

Figure captions and table headings are grammatically correct and generally clear, with appropriate technical language. However, some legends are too brief and could include interpretive statements to highlight key findings. In supplemental figures, small or missing axis labels (e.g., "% kcal") may reduce accessibility. Enhancing label visibility and adding contextual notes would improve clarity for broader audiences.

4. Ethical Considerations and Data Availability

One suggestion for improvement is to more explicitly cite the use of the Open Food Facts (OFF) platform. Although the source is mentioned, a proper bibliographic citation would strengthen the traceability of the dataset. In addition, while the formatting

appears consistent with PLOS ONE style, it is not possible to fully verify this within the manuscript text without access to the reference list in structured format (e.g., EndNote or Zotero output). A final check during typesetting is recommended.

Mentioned References

1. Fazzino, T.L., K. Rohde, and D.K. Sullivan, *Hyper-Palatable Foods: Development of a Quantitative Definition and Application to the US Food System Database.* Obesity (Silver Spring), 2019. **27**(11): p. 1761-1768.

2. Monteiro, C.A., et al., *Ultra-processed foods: what they are and how to identify them.* Public Health Nutr, 2019. **22**(5): p. 936-941.

3. Sutton, C.A., et al., *Ultraprocessed, hyper-palatable, and high energy density foods: Prevalence and distinction across 30 years in the United States.* Obesity (Silver Spring), 2024. **32**(1): p. 166-175.

4. Fazzino, T.L., C. Summo, and A. Pasqualone, *The Italian food environment may confer protection from hyper-palatable foods: evidence and comparison with the United States.* Front Nutr, 2024. **11**: p. 1364695.

5. Dilsaver, D., et al., *Hyper-palatable foods in elementary school lunches: Availability and contributing factors in a national sample of US public schools.* PLoS One, 2023. **18**(2): p. e0281448.

6. Fazzino, T.L., et al., *Meal composition during an ad libitum buffet meal and longitudinal predictions of weight and percent body fat change: The role of hyper-palatable, energy dense, and ultra-processed foods.* Appetite, 2021. **167**: p. 105592.

7. Snelson, M., et al., *Processed foods drive intestinal barrier permeability and microvascular diseases.* Sci Adv, 2021. **7**(14).

8. Atkins, D., et al., *Grading quality of evidence and strength of recommendations.* BMJ, 2004. **328**(7454): p. 1490.

9. Chalmers, I., et al., *How to increase value and reduce waste when research priorities are set.* Lancet, 2014. **383**(9912): p. 156-65.

10. Jackson, R., et al., *The GATE frame: critical appraisal with pictures.* Evid Based Med, 2006. **11**(2): p. 35-8.
