# Supplementary material for: Examination of hyper-palatable foods and their nutrient characteristics using globally crowdsourced data
Source: PLoS One. 2025 Jun 6;20(6):e0325479. doi: 10.1371/journal.pone.0325479 (PMC12143524; doi:10.1371/journal.pone.0325479)
Supplement: Attachment — Submitted filename: R&R Response to Reviewers -HPF Global 1-28-25 FINAL.docx [file pone.0325479.s005.docx]

Thank you for the opportunity to revise and resubmit this manuscript to *PLOS One*. Revisions were made based on reviewer feedback and we believe this has resulted in a stronger manuscript. An item-by-item response is presented below. Reviewer comments are in blue text and our responses are in black text.

Reviewer 1

Abstract:

The abstract is concise and informative. It clearly states the study’s purpose, methodology, key findings, and implications. However, based on the information the authors have provided, it seems that the comparison between HPF and UPF is a significant part of the research. Therefore, it would be beneficial to briefly mention UPF in the abstract to highlight the comparison between HPF and UPF as a key aspect of the research.

We included a statement regarding the HPF and UPF overlap findings in the abstract as recommended.

Introduction
2.1 While the introduction provides a good overview of HPF and their association with obesity risk, it could be strengthened by expanding on the global prevalence of HPF and the need for more research in this area.

We appreciate this point and have elaborated on the likely availability of HPF in the global food environment. However, in the introduction we primarily addressed HPF availability through the established/known role of multi-national food companies that originated in the US and expanded their reach globally, likely disseminating HPF globally. The only other empirical study that has been conducted on HPF availability beyond the US was conducted in Italy, and we site this manuscript in the introduction (p 3). We also included in the introduction the prevalence of ultra-processed foods globally, which have been examined more robustly up to this point (p 4). Finally, we now better emphasize the need for conducting research on HPF in the food environments globally given that this is a substantial gap in the literature and that little is known about HPF outside of the US market (p 4).

2.2 The authors should explicitly state the research gap that this study aims to fill, such as the lack of comprehensive research on the global prevalence and characteristics of HPF.

We have added a statement clarifying the research gap and the goals of the current study to address this gap (p 4-5).

Methods
Please provide additional information about the Open Food Facts database is essential for transparency and replicability, including details on data collection and quality assurance procedures.

We have provided further details regarding the Open Food Facts database and our process for downloading the data and quality control procedures, as requested (p 6). Also, for the purpose of transparency and replicability, R code and the dataset used in this study are fully shared in the Open Science Framework (p 7).

Discussion
4.1Please define strengths of your study

We have added a paragraph to the discussion section detailing the strengths of our study (p 22).

4.2 A deeper discussion of UPF, relating it back to the specific findings of the study and highlighting the prevalence of foods classified as both HPF and UPF, would enhance the analysis.

We have added a paragraph to provide a more in depth discussion of UPF in the context of our findings, as requested (p 21).

4.3 The authors should compare the study’s findings with previous research, highlighting any similarities or discrepancies and discussing potential explanations for any differences.

We have added comparisons to the existing prior studies in the literature on HPF; however there was only one prior study that examined HPF availability outside of the US, which we address in the discussion, and one study that compared HPF and UPF (within the US), which we also discuss (p 18).

4.4 It would be helpful to discuss the implications of the findings for public health interventions.

We have added a paragraph discussing the potential implications of our findings for public health interventions in the conclusions and implications section, as requested (p 23).
