# Supplementary material for: Examination of hyper-palatable foods and their nutrient characteristics using globally crowdsourced data
Source: PLoS One. 2025 Jun 6;20(6):e0325479. doi: 10.1371/journal.pone.0325479 (PMC12143524; doi:10.1371/journal.pone.0325479)
Supplement: Attachment — Submitted filename: R&R2 Response to Reviewers -HPF Global 5-2-25 FINAL.docx [file pone.0325479.s006.docx]

Thank you for the opportunity to revise and resubmit this manuscript to *PLOS One*. Revisions were made based on reviewer feedback and we believe this has resulted in a stronger manuscript. An item-by-item response is presented below. Reviewer comments are in blue text and our responses are in black text.

Reviewer 2

A few aspects could be strengthened to improve clarity and interpretability. First, the conceptual distinction between HPF and UPF—though central to the manuscript—would benefit from clearer explanation earlier in the introduction or discussion. This would help readers unfamiliar with the field better contextualize the comparative findings.

We have added a more detailed description of the distinction between HPF and UPF in the introduction section as recommended (p 5).

Second, while the dataset is extensive, the representativeness of country-level comparisons remains limited. A more detailed discussion of data coverage across countries (e.g., imbalances in product counts, potential input bias, or contributor type) would help interpret the generalizability of results.

We appreciate this point and have added a description of data coverage across countries to the methods section (p 9-10). We incorporated the considerations of data coverage into our discussion of the findings (p 19, 22).

Although the manuscript acknowledges the limitations of working with a crowdsourced database, highlighting a few critical patterns from the supplemental figures in the main text—particularly for nutrient composition—could improve synthesis.

We have added some text descriptions of the supplemental figures, with a focus on nutrient composition, to the main results as recommended (p 12).

The analysis of HPF–UPF overlap is a novel strength of the study and could be more prominently presented in the Results section.

We appreciate the suggestion; however we present the HPF-UPF overlap results in order corresponding with the order of our aims, wherein the overlap aim was last. Therefore, we prefer to retain the current presentation of the results to maintain consistency with study aims.

Finally, while the manuscript is written in standard English, minor stylistic improvements in phrasing and paragraph transitions could enhance readability, particularly in the Results and Discussion.

We have conducted thorough proofreading, attending to stylistic improvements that can better facilitate readability in the results and discussion sections as recommended.
